# Early Detection and Diagnosis of Neonatal Intrahepatic Cholestasis Caused by Citrin Deficiency Missed by Newborn Screening Using Tandem Mass Spectrometry

**DOI:** 10.3390/ijns4010005

**Published:** 2018-01-16

**Authors:** Hiroko Shigetomi, Toju Tanaka, Masayoshi Nagao, Hiroyuki Tsutsumi

**Affiliations:** 1 Department of Pediatrics and Clinical Research, National Hospital Organization Hokkaido Medical Center, 5-7 Yamanote, Nishi-ku, Sapporo 063-0005, Japan; 2 Department of Pediatrics, Sapporo Medical University School of Medicine, S1W16, Chuo-ku, Sapporo 060-8543, Japan

**Keywords:** *SLC25A13*, amino acid ratio, citrullinemia, latent liver dysfunction, mitochondrial aspartate-glutamate carrier

## Abstract

Citrullinemia is the earliest identifiable biochemical abnormality in neonates with intrahepatic cholestasis due to a citrin deficiency (NICCD) and it has been included in newborn screening panels using tandem mass spectrometry. However, only one neonate was positive among 600,000 infants born in Sapporo city and Hokkaido, Japan between 2006 and 2017. We investigated 12 neonates with NICCD who were initially considered normal in newborn mass screening (NBS) by tandem mass spectrometry, but were later diagnosed with NICCD by DNA tests. Using their initial NBS data, we examined citrulline concentrations and ratios of citrulline to total amino acids. Although their citrulline values exceeded the mean of the normal neonates and 80% of them surpassed +3 SD (standard deviation), all were below the cutoff of 40 nmol/mL. The ratios of citrulline to total amino acids significantly elevated in patients with NICCD compared to the control. By evaluating two indicators simultaneously, we could select about 80% of patients with missed NICCD. Introducing an estimated index comprising citrulline values and citrulline to total amino acid ratios could assure NICCD detection by NBS.

## 1. Introduction

Citrin is an aspartate-glutamate carrier found in the mitochondrial membrane and a deficiency was initially found to cause adult-onset type II citrullinemia (CTLN2; OMIM #603471) [1]. Citrin is encoded by the *SLC25A13* gene (cytogenic location; 7q21.3) and its deficiency can manifest in newborns as neonatal intrahepatic cholestasis (NICCD; OMIM #605814) [2,3,4,5]. Since molecular diagnosis became feasible owing to the discovery of prevalent mutations in the *SLC25A13* gene in Japan and East Asia [6,7,8], the clinical features are expanding in other pathogenic states in addition to CTLN2 and NICCD. Failure to thrive and dyslipidemia caused by citrin deficiency (FTTDCD) is another recognized stage of the disease that is characterized by retarded growth and fatty liver in childhood [9]. In the second or later decades, some individuals with citrin deficiency develop CTLN2 with liver dysfunction that is severe enough to require a liver transplantation [10]. The variety of symptoms associated with a lifelong citrin deficiency suggests a need for early diagnosis and treatment to prevent morbidity [11,12].

The symptoms of NICCD are small size for gestational age, prolonged cholestatic jaundice, and failure to thrive in infancy. Laboratory findings include elevated transaminases, hypoproteinemia, and decreased coagulation activity, all suggesting latent liver dysfunction. Galactosemia and multiple amino acidemias—including those of citrulline, methionine, arginine, threonine, and tyrosine—are associated with worsening liver functions after birth. These abnormalities, especially elevated citrulline and galactose, can be detected by NBS but with very low sensitivity [13,14]. Tamamori et al. reported that the first biochemical abnormality detected after birth was citrullinemia and that 95% of patients had over +2 SD of the mean of the neonatal population [15]. Although tandem mass spectrometry has been used for NBS across Japan, the rates of detecting NICCD based on citrulline value have not increased. Most patients are flagged as normal because citrulline is below the screening cutoff at the time. The same cutoff needs to suit both citrullinemia type 1 (CTLN1; also known as arginosuccinate synthetase deficiency) and NICCD if only citrulline is used as the marker. As a result, most patients with NICCD are missed, and overt clinical symptoms then develop later in infancy. To improve newborn screening for citrin deficiency, we surveyed the findings of NBS by tandem mass spectrometry from patients with missed NICCD and investigated biochemical indicators that could lead to a definitive diagnosis.

## 2. Materials and Methods

### 2.1. Newborn Screening Program

Tandem mass screening for neonates within seven days of age started in 2006 in Sapporo (the capital of Hokkaido), and in other areas of Hokkaido in 2012. The Sapporo City Institute of Public Health has implemented NBS, which enabled the analysis of 12 amino acids including citrulline by tandem mass spectrometry. Galactose was measured in the same samples using fluorometric assays. Although citrulline was originally used to detect CTLN1 in NBS, it has been concomitantly applied to detect NICCD in screening panels. The screening cutoff was set at 40 nmol/mL, which was equal to +9.4 SD above the mean of the neonatal population (mean, 11.7; SD, 3).

### 2.2. Patients

Thirteen patients (male, *n* = 8; female, *n* = 5) were referred to our institution for investigation including genetic analyses for suspected NICCD between April 2006 and February 2017. All of them underwent NBS within seven days of birth. Only one boy (patient no. 13) had hypercitrullinemia above the 40 nmol/mL cutoff. However, values for arginine, methionine, tyrosine and galactose were below the cutoff at the first and second examinations. No abnormalities were initially found in 12 neonates (male, *n* = 7; female, *n* = 5) who were labeled as normal. They were referred to us for further diagnosis including DNA testing after the onset of prolonged icterus, white stool, hepatomegaly, and poor weight gain associated with liver dysfunction, at the age of one month or older. A diagnosis of NICCD was confirmed by mutation analysis of the *SLC25A13* gene as well as clinical and laboratory findings (Table 1).

### 2.3. Mutation Analysis

We extracted DNA from peripheral blood cells using a DNA purification kit and exons containing target mutations were amplified using PCR primers as described [7]. The 11 targeted mutations described by Kikuchi et al. [16] (851del4, IVS11+1G>A, 1638ins23, S225X, IVS13+1G>A, IVS16ins3kb, 1800ins1, R605x, E601X, E601K, and L598R) were screened by PCR-RFLP followed by agarose gel electrophoresis and confirmed by direct sequencing. If a mutation was undetectable using this method, entire exons and their boundaries were sequenced to search for infrequent mutations. Parents of patients underwent DNA testing of citrin deficiency to determine parental carrier status.

### 2.4. Statistical Analysis

Statistically significant differences between the sample group and the neonatal population in Sapporo City were assessed using two-sided Z-tests. *p* < 0.005 indicated a statistically significant difference. Data were statistically analyzed using Excel 2016 (Microsoft Corporation, Redmond, WA, USA).

### 2.5. Ethics

The Ethics Committee at Hokkaido Medical Center approved this study (No. 25-2-1, 25 February 2013). Written informed consent was obtained from the guardians of all neonates.

## 3. Results

### 3.1. Amino Acid Analysis at Initial Screening

We retrospectively surveyed citrulline values at the first NBS of 13 patients with NICCD (Table 1). Only one patient (no. 13) had a citrulline value that exceeded the cutoff (74.5 μM; +20.9 SD), immediately leading to a diagnosis of NICCD. All others were deemed normal, because citrulline was below the cutoff. They exceeded the mean of the normal neonates and 80% of them surpassed +3 SD. If the cutoff was reduced to 26.7 μM (mean +5 SD), five neonates would have been flagged as positive at the first screening. However, this would generate an excessive number of false-positive samples (~0.54% of the neonatal population), and an additional screening would become inefficient and costly.

**Figure 1:**
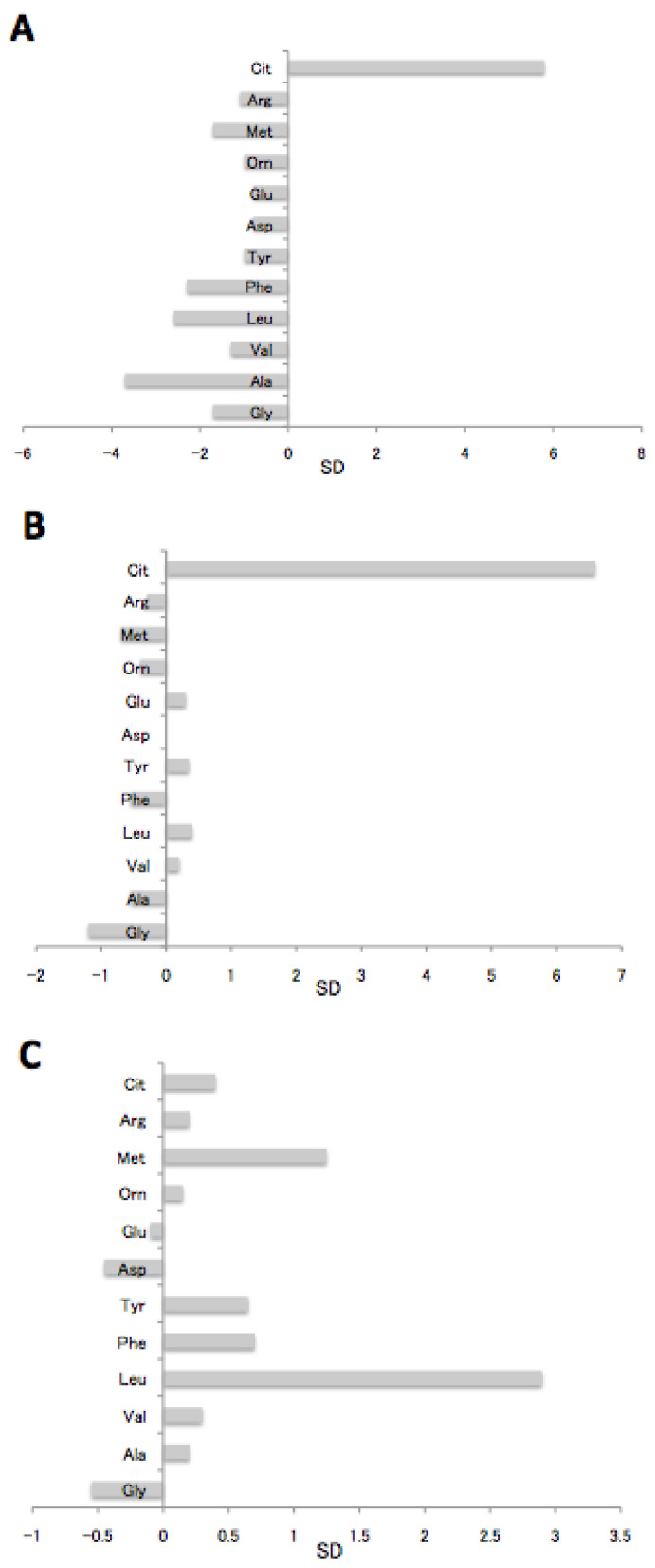

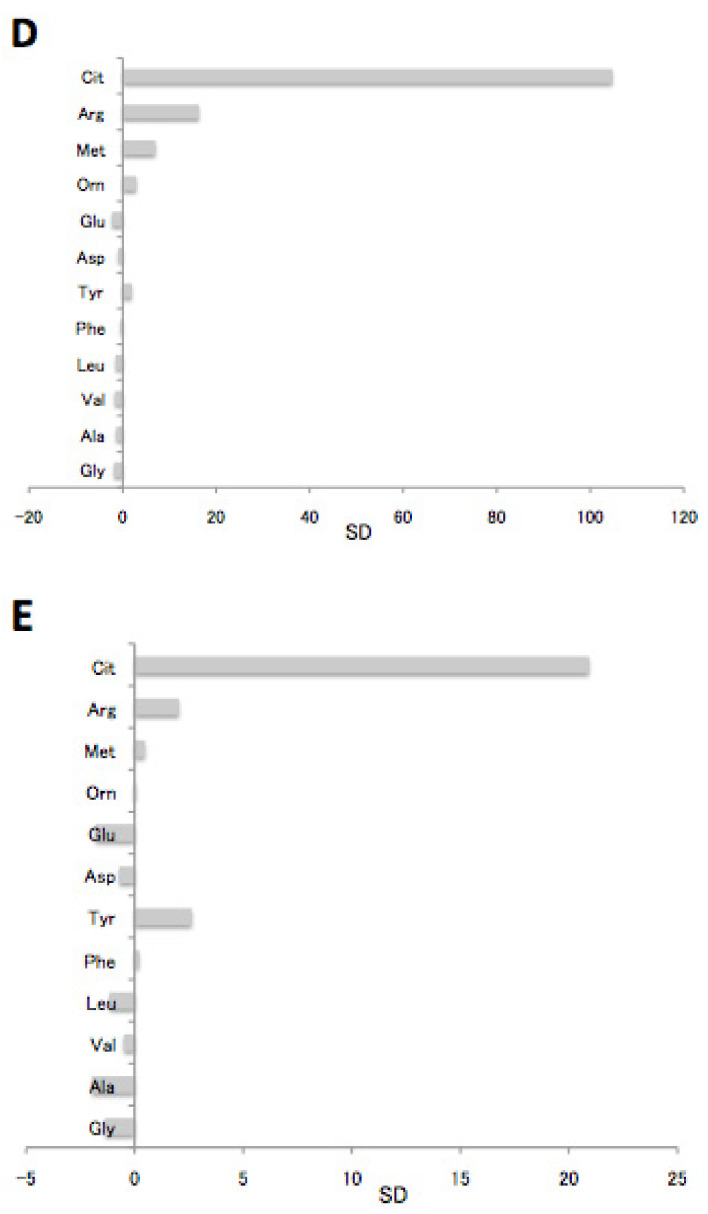
Characteristics of aminograms between patients with missed NICCD and one patient who was NBS-positive. The zero and numbers indicate the average and SD of the neonatal population (*n* = 16360). A, B, C, D, and E: patients 2, 3, 9 (day 5), 9 (day 60), and 13 (NBS positive), respectively. Cit, citrulline; Arg, arginine; Met, methionine; Orn, ornithine; Glu, glutamic acid; Asp, aspartic acid; Tyr, tyrosine; Phe, phenylalanine; Leu, leucine+isoleucine, Val, valine; Ala, alanine; Gly, glycine.

We therefore analyzed the representative aminograms of the neonates with missed NICCD at the first NBS to identify their characteristics. The concentrations of all amino acids other than citrulline were between mean −0.5 SD and mean −4 SD in patient no. 2. Only citrulline was increased (mean +5.8 SD), but remained below the cutoff (Figure 1A). Patient no. 3 also had a relative increase in citrulline when most amino acids remained in the range of −1 SD to +1 SD (Figure 1B). These patients are difficult to flag at the first NBS using citrulline as a specific marker of NICCD and the present cutoff. However, a relative increase in citrulline compared with other amino acids would help to identify early amino acid changes.

The aminogram of patient no. 9 (Figure 1C) showed no abnormality suggesting NICCD on postpartum day 5, but citrulline, arginine, and methionine increased considerably along with the appearance of various symptoms by day 60 (Figure 1D). On the other hand, the typical amino acid profile of NICCD, namely significantly elevated citrulline and mildly or slightly increased tyrosine, arginine, and methionine, was identified by NBS in patient no. 13 (Figure 1E). These results suggest that a large change in the amino acid profile would occur in neonatal period depending on the case.

### 3.2. Screening for Citrin Deficiency Based on Citrulline Values and Relative Increases

Table 2 summarizes the aminograms of initial NBS of 12 patients with NICCD who were missed in the initial NBS. The means of all tested amino acids were statistically compared with those of the general neonatal population using two-sided Z-tests. Citrulline was the most significantly elevated. Glutamic acid and methionine also showed statistically significant differences compared to the control. Although arginine, methionine, and tyrosine have been thought to increase in patients with symptomatic NICCD [17], such changes were not evident in their initial aminograms.

We then evaluated the relative increase in citrulline (Table 3). The sum of all 12 amino acid concentrations in NBS (indicated as tAA) did not differ between patients with NICCD and the general neonatal population. We calculated the ratio of citrulline to tAA, and compared it between two groups. The citrulline/tAA ratio was significantly elevated in patients with NICCD compared to the control.

Table 4 shows the citrulline concentrations and the citrulline/tAA ratios of 13 patients in this study. The citrulline/tAA ratio was highest in patient no. 13 (0.059), who screened positive. At a cutoff of 0.01 (mean +3 SD), 10 neonates with missed NICCD became positive. We then set trial cutoff values of mean +5 SD and mean +3 SD for citrulline and citrulline/tAA, respectively. Five of 12 missed neonates who met both indices were flagged as having NICCD, suggesting that simultaneous use of these parameters can accurately screen for NICCD.

### 3.3. Estimated NICCD Index

We designed the NICCD index to estimate the likelihood of detecting NICCD in the first NBS specimen. It consists of absolute and relative increases in citrulline. The former is the actual concentrations of citrulline above 40 nmol/mL (the current cutoff for CTLN1 and NICCD) or 26.7 nmol/mL (the mean +5 SD), and the latter comprises citrulline/tAA ratio above 0.01 (the mean +3 SD). We scored and classified each value according to total scores of 4 (definitive), 3 (probable), and 1–2 (possible) (Table 4). One NBS-positive patient (no. 13) scored 4, which was compatible with a diagnosis of NICCD. CTLN1 also needs to be considered in patients with a score of 4 while referring to the clinical course. In applying this index to neonates with missed NICCD, we flagged five patients each as having probable and possible NICCD.

### 3.4. Mutation Spectrum of Patients with NICCD

Genetic testing revealed *SLC25A13* gene mutations (3 homozygotes and 10 compound heterozygotes) in 13 infants (Table 1). We found five known mutations (851del4, IVS11+1G>A, S225X, IVS13+1G>A and E601X) and a missense variant (Y504C, c.1511A>G), which is predicted to be damaging through PolyPhen-2 and SHIFT (rs 777414201 SNP). The most frequently detected was IVS11+1G>A (14 alleles, 54% of disease alleles), followed by 851del4 (7 alleles, 27%). These two mutations comprised 81% of the mutated alleles.

## 4. Discussion

The reported frequency of homozygotes or compound heterozygotes for *SLC25A13* mutations in Japan is 1/17,000 and the carrier rate is 1/65 [8]. In addition, Shigematsu et al. reported that the prevalence of NICCD would also be 1/17,000 to 1/34,000 among the Japanese population [18]. Since almost 600,000 babies were born in Sapporo city and Hokkaido between 2006 and 2017, 18 to 35 should have NICCD. However, only one neonate was positive for NICCD according to the NBS during this period. Twelve patients in the present study were identified only after becoming clinically symptomatic. These results suggested that the sensitivity of the present mass screening to detect NICCD in neonates is quite low.

The present cutoff for citrulline was originally set to detect both CTLN1 and NICCD. However, this value is not appropriate for detecting NICCD since the range of 12 missed patients was 12.6 to 31.1 nmol/mL, which was well below the cutoff. Yet, setting a lower cutoff value would increase the false-positive rate. Tamamori et al. reported the importance of total amino acid values and relative increases in citrulline among patients who were negative in NBS using an HPLC system [15]. We therefore compared increases in citrulline to those of other amino acids based on the characteristic amino acid profiles of the patients with missed NICCD. By evaluating citrulline and Cit/tAA ratio simultaneously, NICCD can be detected with higher sensitivity by tandem mass spectrometry. Nearly 80% of missed patients will be picked up based on the result of this study.

To detect NICCD using a single metabolic marker and a single sample is quite difficult. Wang et al. have suggested additional or second-tier screening tests [19]. Pathogenic metabolic changes due to NICCD develop during the next few weeks after birth. Therefore, most individuals with NICCD become symptomatic at about one month after birth or later. If several markers are prepared to flag suspected NICCD at the time of the one-month health check, a physician or pediatrician can easily refer an infant with symptoms to a specialist. We therefore devised the estimated NICCD index based on the present data. Automatic calculation of the index in NBS will select latent asymptomatic NICCD more precisely and efficiently. Delayed diagnosis and treatment of NICCD imposes a burden upon patients and their families, and can lead to unnecessary investigation and occasionally prolonged hospitalization. Application of the estimated NICCD index to such individuals helps decision-making about the need for an urgent clinical survey.

It is now feasible to diagnose a citrin deficiency by genetic testing, since six major mutations explain almost 90% of pathogenic alleles among the Japanese population. In addition, searching infrequent mutations in exon 17 means that >95% can be covered, leading to an accurate and prompt diagnosis of citrin deficiency [16,20]. Our mutation analysis of 13 neonates with NICCD detected IVS11+1G>A the most frequently as it comprised >50% of disease alleles. This predominance of IVS11+1G>A has not been observed in other regions of Japan. The characteristic features of the mutation spectrum of *SLC25A13* might be related to geographic and historical aspects of Hokkaido. People started to migrate from the Japanese mainland to various parts of Hokkaido (the most northern district) during the 19th century. It may be that individuals who were asymptomatic homozygous or heterozygous carriers of IVS11+1G>A were significantly included in the population.

We concluded that with increased awareness of NICCD among physicians and pediatricians at one-month health checks, re-evaluation of neonatal mass screening results using the estimated NICCD index would prevent morbidity arising during infancy and progression to FTTDCD and CTLN2 over time.

## Figures and Tables

**Table 1:** Characteristics of patients with NICCD

Patient No.	Sex	Cit (nmol/mL) *	Onset (Month)	Allele 1	Allele 2	Initial Symptoms
1	F	19.4	3	IVS11+1G>A	IVS13+1G>A	Poor weight gain, icterus, white stool, developmental delay
2	F	29.2	1	IVS11+1G>A	S225X	Poor weight gain, icterus, white stool
3	M	31.1	1	IVS11+1G>A	IVS11+1G>A	Icterus, white stool
4	M	23.2	4	851del4	IVS11+1G>A	Icterus, hepatomegaly
5	F	29	4	IVS11+1G>A	Y504C	Hepatomegaly
6	M	26.8	1	851del4	IVS11+1G>A	Icterus, anemia
7	M	18.9	1	851del4	IVS11+1G>A	Poor weight gain, icterus, white stool
8	F	26	1	IVS11+1G>A	IVS11+1G>A	Poor weight gain
9	M	13	1	IVS11+1G>A	E601X	Poor weight gain, icterus
10	M	29.7	2	851del4	851del4	White stool
11	M	12.6	4	IVS11+1G>A	IVS11+1G>A	Poor weight gain, icterus
12	F	19.7	2	851del4	IVS13+1G>A	Icterus
13	M	74.5	0 (NBS+)	851del4	IVS11+1G>A	None

* Citrulline (Cit) cutoff: 40 nmol/mL.

**Table 2:** Aminogram of missed patients with NICCD.

AA (nmol/mL)	NICCD (*n* = 12)			Neonatal Population		*p* ^a^
				(*n* = 16,360)		
	Mean	SD	Range	Mean	SD	
Glycine	324.1	125.8	170.3–644	362.3	107.3	0.581
Alanine	282.7	118.1	123–508	280.8	82.7	0.938
Valine	102.7	33.4	56.5–140.6	108	27.1	0.497
Leucine+Isoleucine	193.2	62.9	97.6–294.6	182.8	35.2	0.281
Phenylalanine	46.9	10.5	29.5–70.8	48.1	8.9	0.64
Tyrosine	129.4	51.2	59.4–233.3	103.4	37.3	0.016
Aspartic acid	53.2	19.7	26.8–83.5	41.7	17.3	0.021
Glutamic acid	352.2	90.8	239.9–519.5	296.5	60.9	0.001
Ornithine	119.5	51.8	56.3–231.8	109.9	43.3	0.443
Methionine	17.7	5.3	11.6–28.2	21.5	4.5	0.004
Arginine	14.8	6.6	7.7–25.9	12.6	5.6	0.173
Citrulline	23.2	6.4	12.6–31.1	11.7	3	<0.001

^a^ Based on two-sided Z-test; AA, amino acid.

**Table 3:** Comparison of total amino acids and citrulline/total amino acids between missed patients with NICCD and controls

Marker	NICCD (*n* = 12)			Controls (*n* = 16360)		*p* *
	Mean	SD	Range	Mean	SD	
tAA	1667	464	990–2686	1587	308	0.18
Cit/tAA	0.015	0.006	0.008–0.029	0.007	0.001	<0.001

Controls: age-matched neonatal population. * Two-sided Z-test. tAA, total amino acid.

**Table 4:** Estimated NICCD index.

Patient	Cit (nmol/mL)	Cit/tAA	Score
1	19.4	0.009	0
2	29.2	0.029	3
3	31.1	0.021	3
4	23.2	0.015	1
5	29	0.011	3
6	26.8	0.016	3
7	18.9	0.018	1
8	26	0.015	1
9	13	0.008	0
10	29.7	0.015	3
11	12.6	0.011	1
12	19.7	0.011	1
13 (NBS+)	74.5	0.059	4
	Cit (Screening Cutoff)	Cit	Cit/tAA
Cutoff	40 (+9.4 SD)	26.7 (+5 SD)	0.01 (+3 SD)
Score	3	2	1
Judgement	Definitive	4	
	Probable	3	
	Possible	1–2	
